# RETRACTION: Use of Local Wound Infiltration in Open Hepatectomy to Reduce Wound Pain: A Systematic Review and Meta‐Analysis

**DOI:** 10.1111/iwj.70305

**Published:** 2025-03-06

## Abstract

**RETRACTION:** MengQ.‐Qi. , ZhaoQ.‐C. , and HouD.‐N. , “Use of Local Wound Infiltration in Open Hepatectomy to reduce Wound Pain: A Systematic Review and Meta‐Analysis,” International Wound Journal 20, no. 9 (2023): 3760–3767, 10.1111/iwj.14271.37287429 PMC10588336

The above article, published online on 07 June 2023, in Wiley Online Library (http://onlinelibrary.wiley.com/), has been retracted by agreement between the journal Editor in Chief, Professor Keith Harding; and John Wiley & Sons Ltd. Following an investigation by the publisher, all parties have concluded that this article was accepted solely on the basis of a compromised peer review process. The editors have therefore decided to retract the article. The authors did not respond to our notice regarding the retraction.

**RETRACTION:** Q.‐Qi. Meng , Q.‐C. Zhao , and D.‐N. Hou , “Use of Local Wound Infiltration in Open Hepatectomy to reduce Wound Pain: A Systematic Review and Meta‐Analysis,” International Wound Journal 20, no. 9 (2023): 3760–3767, 10.1111/iwj.14271.37287429 PMC10588336

The above article, published online on 07 June 2023, in Wiley Online Library (http://onlinelibrary.wiley.com/), has been retracted by agreement between the journal Editor in Chief, Professor Keith Harding; and John Wiley & Sons Ltd. Following an investigation by the publisher, all parties have concluded that this article was accepted solely on the basis of a compromised peer review process. The editors have therefore decided to retract the article. The authors did not respond to our notice regarding the retraction.

